# Effect of Vertebral Fracture on Auxological Profiles of Children Undergoing Acute Lymphoblastic Leukemia Treatment

**DOI:** 10.3389/fped.2021.686128

**Published:** 2021-06-16

**Authors:** Moon Bae Ahn, Seongkoo Kim, Won Kyoung Cho, Jae Wook Lee, Min Ho Jung, Nack-Gyun Chung, Bin Cho, Byung-Kyu Suh

**Affiliations:** Department of Pediatrics, College of Medicine, Catholic University of Korea, Seoul, South Korea

**Keywords:** vertebral fracture, osteoporosis, childhood ALL, bone mass density, ALL treatment, auxological profile, pediatric malignancy

## Abstract

**Background:** Acute lymphoblastic leukemia (ALL) is the most common pediatric malignancy, and children with ALL often experience skeletal morbidity such as vertebral fractures (VF) during and after ALL treatment. Among various treatment-associated factors that affect growth pattern, the presence of VF might trigger growth impairment.

**Objective:** This study aimed to investigate the overall VF incidence following childhood ALL treatment and examined the association of VF with growth.

**Methods:** Children diagnosed with ALL whose treatment was completed between 2 and 15 years of age and who were screened with lateral thoracolumbar spine radiographs were enrolled. Clinical data, including anthropometric parameters were obtained at leukemia diagnosis (LD), treatment completion (TC), and 12 months following TC while VF assessment were obtained at TC and 12 months following TC.

**Results:** In total, 155 children were included, and height status was decreased, whereas weight and BMI status were increased throughout three observational points. VF incidence at TC was 18.7%. Height status were lower in children with VF at LD, TC, and 12 months following TC, while a greater height decline was observed during the treatment period. Age and height status at LD and average glucocorticoid (GC) dose were associated VF incidence at TC. The presence of VF was a significant risk factor of height decline during the treatment period.

**Conclusion:** A substantial number of children experienced VF following ALL treatment completion, and the presence of VF might adversely affect auxological status in children. VF detection by routine surveillance throughout childhood ALL treatment is recommended to try to prevent compromised growth.

## Introduction

Acute lymphoblastic leukemia (ALL) is the most common pediatric malignancy, with improved cure rates owing to an evolved therapeutic regimen and supportive care. Stratification and treatment allocations are based on multiple prognostic factors, including blood count at diagnosis, central nervous system involvement, immunophenotype, cytogenetic, and genomic features while minimal residual disease at specified time point is critical for the response to treatment (1). The current chemotherapy for ALL includes four phases of a step-by-step regimen; however, patients stratified as a high-risk group finally requires peripheral blood stem cell transplantation (1). ALL in childhood or adolescence is related to various endocrinopathies after the cessation of therapy, which may persist for several years (2). Most importantly, skeletal morbidities due to osteoporosis and vertebral fractures (VFs) caused by impaired mineralization are common endocrinologic complications that occur during and after ALL treatment (3–5).

Children with chronic disease are at risk for developing bone problem (6, 7). VFs are prominent sequelae of bone demineralization and osteoporotic change and are frequently asymptomatic, making them difficult to identify (8). Increased bone resorption at ALL diagnosis due to osteoclast-activating cytokines, caused by the leukemic disease and the prolonged use of osteotoxic drugs, such as glucocorticoids (GC) and methotrexate, contribute to VF incidence and an insufficient bone mineral accrual (9, 10). According to the Canadian Steroid-Associated Osteoporosis in the Pediatric Population study, a 4-years cumulative incidence of VFs in ALL children could be as high as 26.4%; therefore, routine surveillance for VF is critical (8). Additionally, incidental VF is a risk factor for major height decline in patients with ALL in the early treatment period, and the number and severity of VF are directly related to the compromised height (11).

To date, the growth impairment in ALL has been assumed to be caused by the direct effect of chemotherapeutic agents and cranial irradiation dosage. However, there is a lack of evidence regarding the effect of VF on the long-term auxological profile of children with ALL. Although factors leading to compromised adult height or the occurrence of increased body mass index (BMI) after ALL treatment need to be elucidated, low bone mineral density (BMD), followed by VF affecting height and weight parameters, could be the principal determinant of post-treatment growth pattern in children with ALL. Therefore, this study aimed to investigate the overall incidence of VF following childhood ALL treatment and to examine the association of VF with growth status.

## Methods

### Subjects

**Figure 1:**
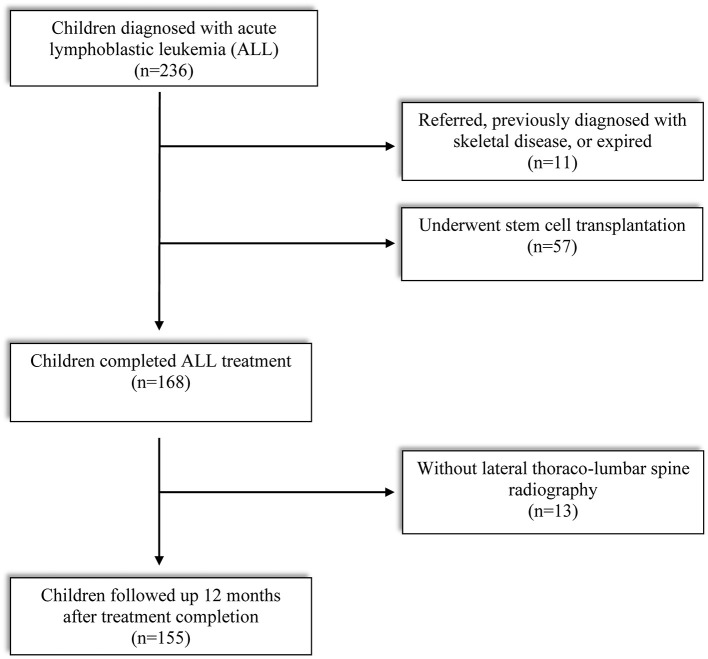
Flow chart of subjects enrolled.

Enrollment criteria of subjects and reasons for exclusion are described in Figure 1. Out of 234 children diagnosed with ALL between 2015 and 2020, finally 155 subjects met the eligibility criteria: (1) treatment was completed (based on our institutional protocol) between 2 and 15 years of age and (2) screened by lateral thoracolumbar (D-L) spine radiography at treatment completion (TC) and 12 months after TC. Children who received peripheral blood stem cell transplantation, transferred from other institution, previously diagnosed with skeletal disease, or expired in the middle of the treatment period were excluded. This study was approved by the Institutional Review Board of the Catholic University of Korea, Seoul St. Mary's hospital (KC21RISI0051). The requirement of informed patient consent was waived because no intervention and further examination was performed.

### Risk Stratification and Treatment of All

In accordance with our uniform institution-based protocol, ALL patients were classified into 4 risk groups of low, standard, high, and very high risk, based on the clinical features of the patient, characteristics of the leukemic blast, and treatment response (12). A key feature of the ALL treatment included risk group based post-Consolidation therapy, with low and standard risk patients receiving one course each of Interim Maintenance and Delayed Intensification, while high and very high patients received two courses of each phase of therapy (12). None of the patients received cranial irradiation during first complete remission. Furthermore, high dose methotrexate and intrathecal methotrexate were administered every 12 weeks during Maintenance phase, in addition to daily 6-mercaptopurine, weekly methotrexate, and monthly pulses of vincristine and steroid (12).

### Clinical Data

Baseline characteristics of the patients, including age, sex, treatment duration, and leukemia classification, were collected. Anthropometric parameters, including height, weight, and BMI, were obtained and assessed at following time points: (1) leukemia diagnosis (LD), (2) TC, and (3) 12 months following TC. Height was measured to the nearest centimeter using a regularly calibrated stadiometer (Harpenden Stadiometer, Holtain®, UK). Weight was determined using a digital scale (Simple Weighing Scale, Cas®, Korea). All anthropometric data were converted to age- and sex-matched standard deviation scores (SDS), using the national growth chart (13). An increase or decrease in height, weight, and BMI, between the assessment points, was expressed as Δ. During the treatment, GC dosing was supervised by pediatric hemato-oncologists according to the protocol, and accumulated doses were converted to prednisolone equivalents, expressed as milligrams per body surface area.

### VF Assessment and Lumbar Spine BMD

VF was diagnosed when a reduction in vertebral height was observed and the reduction was graded, according to the degree of deformity, as mild (20–25%), moderate (25–40%), or severe (>40%) reduction in anterior, middle, and/or posterior heights relative to the same or an adjacent vertebra. D-L spine radiography was used as an imaging tool to detect deformity in 13 vertebral bodies (T5 to L5), and radiographs were obtained at TC and 12 months following TC. The presence of VF was determined by two pediatric endocrinologists using a modified Genant semiquantitative technique (14). The final decision, confirming the presence of a fracture, and thereby resolving any discrepancies in the analyses of the pediatric endocrinologists, was made by a pediatric radiologist.

Lumbar spine bone mineral density (LSBMD) assessment was performed after TC for patients aged >10 years. LSBMD was measured in the anterior-posterior direction (L1–L4) using dual-energy X-ray absorptiometry (DXA) (Horizon W DXA system®, Hologic Corp. USA). A single radiographer, who was blinded to the clinical histories of the patients, was in charge of all BMD measurements. A two-dimensional computation of bone mineral content (g/cm^2^) was determined and converted to SDS, according to age- and sex-matched national normative references (15). Low bone mass (LBM) was defined as LSBMD SDS ≤ −1.0.

### Statistics

Statistical analyses included descriptive analysis of all variables, expressed as either mean ± standard deviation for normally distributed values or median (interquartile range 25%, 75%) for non-normally distributed data. The normality of numerical variables was tested through the Kolmogorov-Smirnov test. Comparison of clinical characteristics between children with and without VF was performed via the independent *t*-test. Univariate and multivariate binomial logistic regression analyses were used to determine the potential risk factors presented with odds ratio (OR) and 95% confidence interval to associate with VF incidence. Univariate and multivariate linear regression analyses were used to determine the potential risk factors presented with beta coefficients (β) and 95% confidence interval to associate with height decline. All statistical analyses were performed using SPSS version 24.0 (IBM Corp®, Armonk, NY, USA).

## Results

### Baseline Characteristics of Subjects

**Figure 2:**
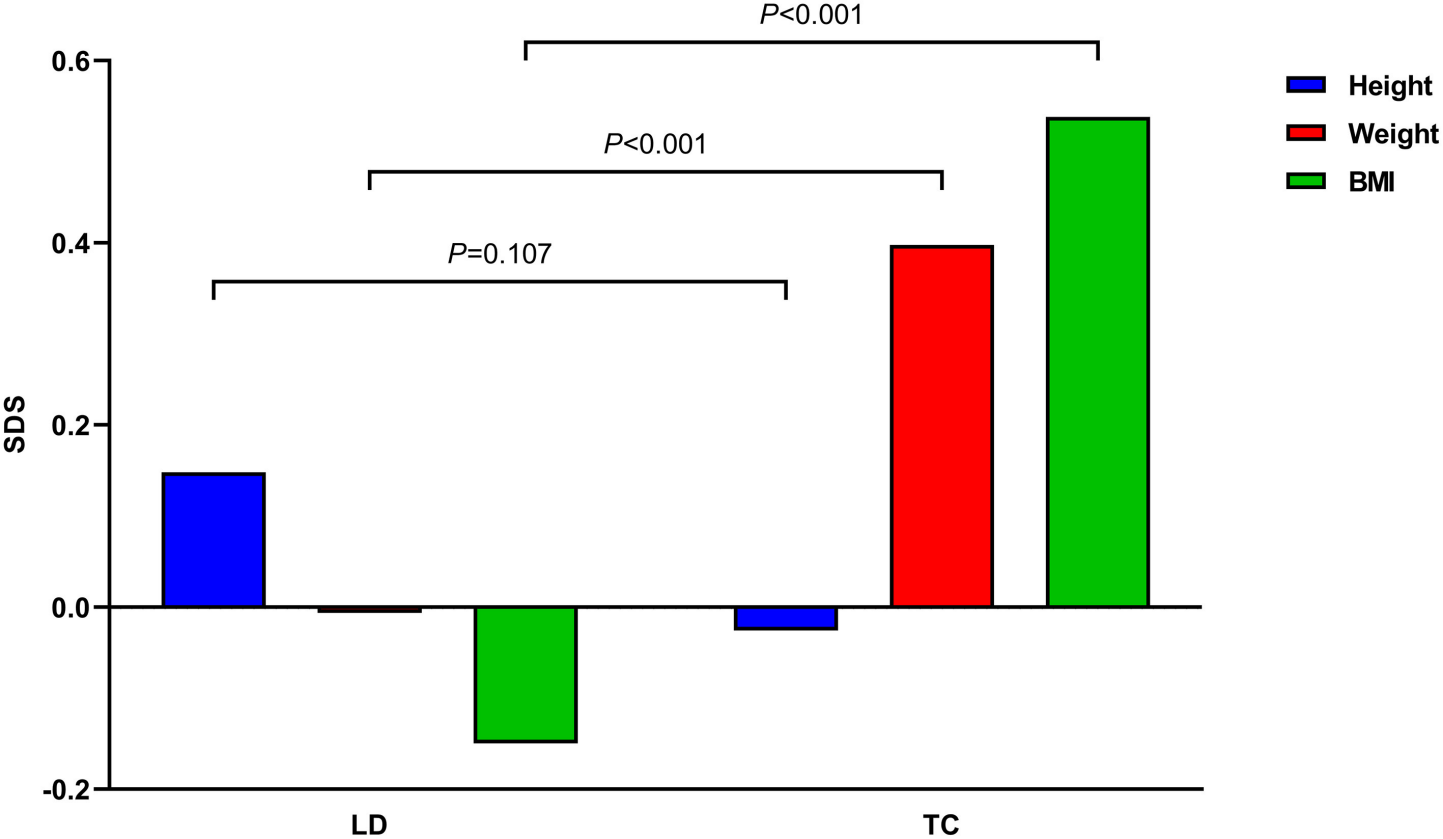
Mean height, weight, and body mass index (BMI) standard deviation scores (SDS) of all children before and after acute lymphoblastic leukemia treatment are demonstrated. *P-*values between times point is denoted.

A total of 155 children with ALL were included in this study (Table 1). The median age was 5.9 years (3.4, 7.9) at LD and 8.1 years (6.3, 10.8) at TC, with median treatment duration of 2.8 years (2.6, 2.9). The number of patients with precursor B-cell ALL was 147 (94.8%), while 48 (30.9%) children were stratified as high-risk and 46 (29.7%) as low-risk patients. None of the patients received prophylactic cranial irradiation in first complete remission. The total accumulated GC dose administered until the end of treatment was 7715.93 ± 1548.27 mg/m^2^. All children after ALL treatment showed decreased height SDS and increased weight and BMI SDS (Figure 2).

**Table 1:** Description of the cohort.

**Clinical parameters**	**Total (*n =* 155)**
Demographic data at ALL diagnosis	
Male, *n* (%)	91 (58.7)
Age, years, median (IQR 25%, 75%)	5.9 (3.4, 7.9)
Leukemia characteristics	
**Classification**, ***n*** **(%)**	
Pre-B-cell	147 (94.8)
T-cell	7 (4.5)
Biphenotype	1 (0.6)
**Risk category**, ***n*** **(%)**	
Low	46 (29.7)
Standard	39 (25.2)
High	48 (30.9)
Very high	22 (14.2)
VF assessment at treatment completion	
VF, *n* (%)	29 (18.7)
Age, years, median (IQR 25%, 75%)	8.1 (6.3, 10.8)
**Tanner stage**, ***n*** **(%)**	
I	93 (60.0)
II	28 (18.1)
III	25 (16.1)
IV	7 (4.5)
V	2 (1.2)
Duration of treatment, years, median (IQR 25%, 75%)	2.8 (2.6, 2.9)
Accumulated GC dose (mg/m^2^)	7715.93 ± 1548.27
**Grading**, ***n*** **(%)**	
Mild	17 (58.6)
Moderate	12 (41.4)
Vertebral level	
Lumbar	19 (65.5)
Thoracic	10 (34.5)
DXA, *n* (%)	46 (29.7)
LSBMD SDS	−0.63 ± 1.01
LBM, *n* (%)	17 (36.9)

*All values are expressed as mean ± standard deviation unless mentioned*.

*ALL, acute lymphoblastic leukemia; DXA, dual-energy x-ray absorptiometry; GC, glucocorticoid; IQR, interquartile range; LBM, low bone mass; LSBMD, lumbar spine bone mineral density; VF, vertebral fractures*.

### Frequency of VF and LBM

The total number of children with VF at TC was 29 (18.7%), and these patients had either mild (17 patients; 58.6%) or moderate (12 patients; 41.4%) deformities. The lumbar vertebrae were more commonly injured (19 patients; 65.5%), while L3 (six patients; 20.7%) was found to be the most frequently injured site. Three children with moderate VF (2 children with T6 and 1 child with L4 fracture) subsequently showed mild deformity on follow-up radiographs after 12 months, although this change did not affect the total number of children with VF. None of the children had aggravated VF during follow-up.

DXA was performed in 46 (29.7%) children, and 17 (36.9%) children were found to have LBM. LSBMD SDS was significantly lower in children with VF than in those without VF (−1.32 ± 0.77 and −0.18 ± 0.92, respectively, *P* < 0.001). Twelve (70.6%) children with VF had LBM, while a cut-off of−0.42 for the LSBMD accounted for sensitivity of 66.7% and specificity of 71.4% in regard with VF incidence (area under the curve, 0.84).

### Anthropometry of Children With and Without VF

The height, weight, and BMI of children with VF were compared with those of children without VF (Table 2). Height SDS was significantly lower in children with VF than in those without VF at LD, TC, and 12 months after TC (−0.19 ± 0.98 vs. 0.23 ± 0.95, *P* = 0.033; −0.59 ± 0.86 vs. 0.11 ± 0.89, *P* < 0.001; −0.54 ± 0.86 vs. 0.18 ± 0.89, *P* < 0.001, respectively). BMI SDS was higher in children with VF compared with those without VF at 12 months after TC (0.98 ± 1.39 vs. 0.26 ± 1.21, *P* = 0.006), whereas it showed no significant difference between the two groups at LD and TC.

**Table 2:** Anthropometric profiles of ALL patients according to follow-up points at leukemia diagnosis, treatment completion, and 12 months after treatment completion with respect to the presence of VF.

	**With VF (*n =* 29)**	**Without VF (*n =* 126)**	***P***
At leukemia diagnosis	−0.19 ± 0.98	0.23 ± 0.95	
Height SDS	−0.15 ± 1.09	0.03 ± 0.95	0.033^*^
Weight SDS	−0.09 ± 1.10	−0.16 ± 1.03	*0.399*
BMI SDS			*0.72*
Between leukemia diagnosis and treatment completion	−0.40 ± 0.43	−0.12 ± 0.63	
ΔHeight SDS	0.53 ± 1.02	0.37 ± 0.81	0.025^*^
ΔWeight SDS	0.96 ± 1.35	0.63 ± 1.15	*0.37*
ΔBMI SDS			*0.172*
At treatment completion	−0.59 ± 0.86	0.11 ± 0.89	
Height SDS	0.39 ± 1.23	0.40 ± 1.06	<0.001^*^
Weight SDS	0.87 ± 1.46	0.46 ± 1.19	*0.955*
BMI SDS			*0.11*
Between treatment completion and 12 months after treatment completion	0.06 ± 0.31	0.06 ± 0.30	
ΔHeight SDS	0.12 ± 0.46	−0.11 ± 0.48	*0.863*
ΔWeight SDS	0.10 ± 0.59	−0.20 ± 0.64	0.018^*^
ΔBMI SDS			0.021^*^
At 12 months after treatment completion	−0.54 ± 0.86	0.18 ± 0.89	
Height SDS	0.51 ± 1.14	0.29 ± 1.09	<0.001^*^
Weight SDS	0.98 ± 1.39	0.26 ± 1.21	*0.327*
BMI SDS			0.006^*^

*All values are expressed as mean ± standard deviation*.

*ALL, acute lymphoblastic leukemia; BMI, body mass index; VF, vertebral fracture; SDS, standard deviation score*.

* *P < 0.05*.

*Bold values are indicated for P < 0.05 (statistically significant). Italic values are indicated for all P-values*.

**Figure 3:**
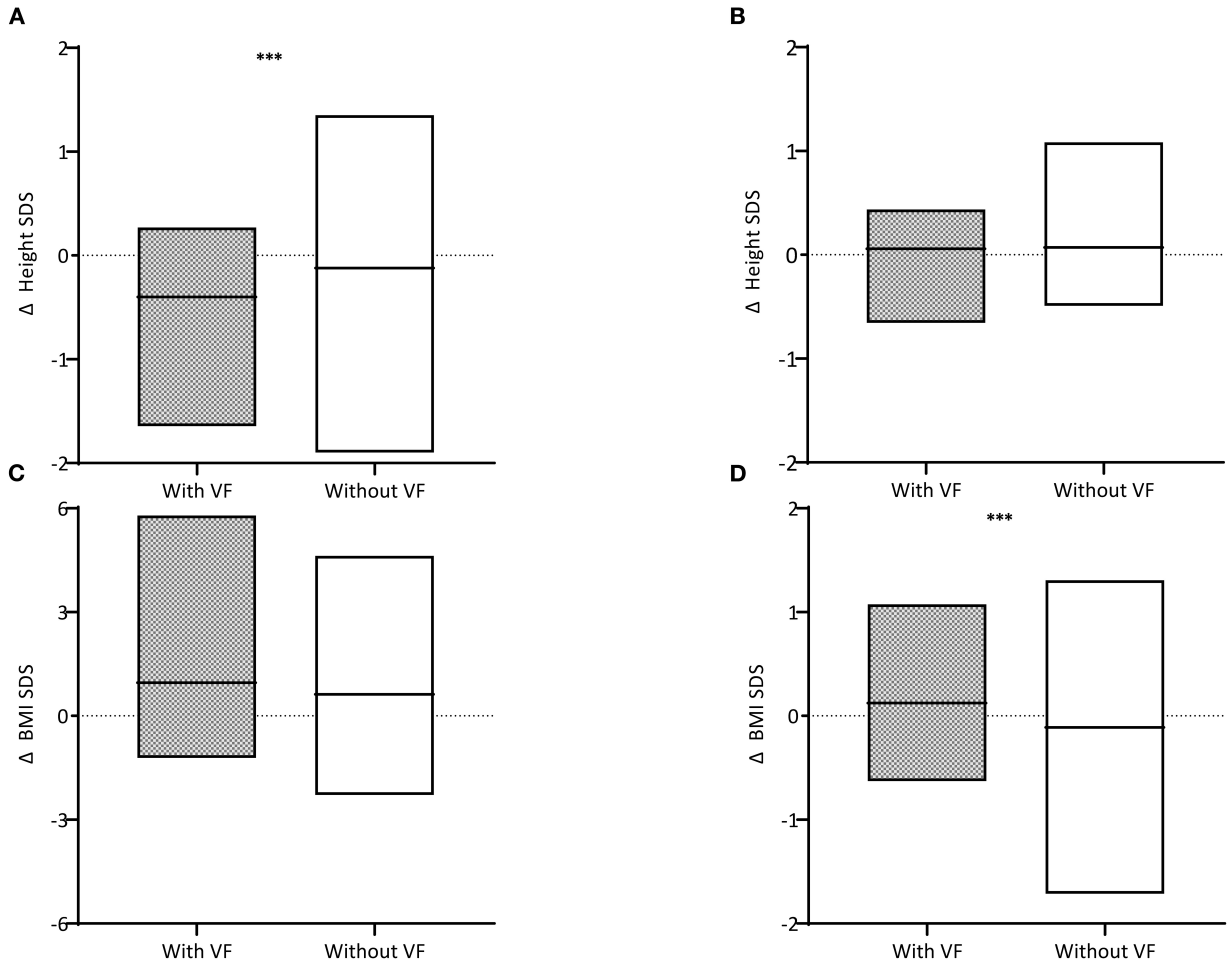
Difference (Δ) in height standard deviation scores (SDS) **(A)** during the treatment period and **(B)** within 12 months after treatment completion and difference in body mass index (BMI) SDS **(C)** during the treatment period and **(D)** within 12 months after treatment completion are described. Children with vertebral fractures (VF) are shown in gray boxes and those without VF in white boxes. The horizontal black lines within the box indicate the mean. Three asterisks indicate for *P* < 0.05.

The differences in height, weight, and BMI status during the treatment period and within 12 months after TC were compared (Table 2 and Figure 3). A greater decline was observed in children with VF than in those without VF (−0.40 ± 0.43 and −0.12 ± 0.63, respectively, *P* = 0.025). Height status showed no significant change for 12 months following TC, and it was not different between children with VF and those without VF (0.06 ± 0.31 and 0.06 ± 0.30, respectively, *P* = 0.863). BMI was increased over the 12-month period following TC in children with VF and decreased in children without VF (0.10 ± 0.59 and −0.20 ± 0.64, respectively, *P* = 0.021) which resulted in weight change in a similar manner (0.12 ± 0.46 and −0.11 ± 0.48, respectively, *P* = 0.018).

### Risk Factors Associated With VF and Height Decline

Binomial logistic regression analyses were performed to determine the risk factors associated with VF incidence (Table 3). Univariate analysis was conducted to assess the following clinical factors: sex, leukemia criteria, risk group, age, treatment duration, GC dose, Tanner stage, and height and BMI profiles at each time point with VF incidence. Age at LD [1.21 [1.06 to −1.38] odds ratio OR [95% confidence interval], *P* = 0.004], treatment duration [5.07 [1.03–24.9], *P* = 0.046], average GC dose [1.68 [1.07–2.62], *P* = 0.024], height SDS at LD [0.61 [0.38–0.97], *P* = 0.036], and height SDS at TC [0.39 [0.23–0.37], *P* < 0.001] were associated with VF incidence. After adjusting for sex, leukemia criteria, treatment duration, and average GC dose, age at LD [1.18 [1.03–1.35], *P* = 0.018] and height SDS at LD [0.61 [0.38–0.96], *P* = 0.034] remained significantly associated with VF incidence in the final multivariate analyses.

**Table 3:** Univariate and multivariate regression analyses of factors associated with the vertebral fracture incidence during the treatment period.

**Risk factors**	**Univariate**			**Multivariate**		
	**OR (95% CI)**	**SE**	***P*-value**	**OR (95% CI)**	**SE**	***P*-value**
Sex	NS					
Leukemia criteria	NS					
Risk group	NS					
Age at leukemia diagnosis	1.21 (1.06–1.38)	0.07	0.004^*^	1.18 (1.03–1.35)	0.24	0.018^*^
Treatment duration	5.07 (1.03–24.9)	0.81	0.046^*^			
Average GC dose	1.68 (1.07–2.62)	0.23	0.024^*^			
Tanner stage at treatment completion	NS					
Height SDS at leukemia diagnosis	0.61 (0.38–0.97)	0.24	***0.036***	0.61 (0.38–0.96)	0.24	0.034^*^
BMI SDS at leukemia diagnosis	NS					
Height SDS at treatment completion	0.39 (0.23–0.67)	0.27	< 0.001^*^			
BMI SDS at treatment completion	NS					

*BMI, body mass index; CI, confidence interval; GC, glucocorticoid; NS, not significant; OR, odds ratio; SDS, standard deviation score; SE, standard error*.

* *P < 0.05*.

*Bold values are indicated for P < 0.05 (statistically significant). Italic values are indicated for all P-values*.

Another univariate regression was conducted to test the effect of VF on height decline during the treatment period (Table 4). Age at LD [0.07 [0.04–0.10] β [95% confidence interval], *P* < 0.001], height SDS at LD [−0.24 [0.15–0.33], *P* < 0.001], and the presence of VF [0.28 [0.03–0.52], *P* = 0.025] were associated with increase in height decline during the treatment period. After adjusting for age at LD and heigh SDS at LD, the presence of VF [0.27 [0.05–0.49], *P* = 0.016] remained significantly associated with increase in height decline in the final multivariate analyses.

**Table 4:** Univariate and multivariate regression analyses of factors associated with height decline during the treatment period.

**Risk factors**	**Univariate**			**Multivariate**		
	**β (95% CI)**	**SE**	***P*-value**	**β (95% CI)**	**SE**	***P-value***
Sex	NS					
Leukemia	NS					
Risk group	NS					
Age at leukemia diagnosis	0.07 (0.04–0.10)	0.02	<0.001^*^			
Treatment duration	NS					
Average GC dose	NS					
Tanner stage at treatment completion	NS					
Height SDS at leukemia diagnosis	−0.24 (0.15–0.33)	0.05	<0.001^*^			
BMI SDS at leukemia diagnosis	NS					
Height SDS at treatment completion	NS					
BMI SDS at treatment completion	NS					
VF	0.28 (0.03–0.52)	0.12	***0.025***	0.27 (0.05–0.49)	0.11	0.016^*^

*β, beta coefficient; CI, confidence interval; NS, not significant; OR, odds ratio; SDS, standard deviation score; SE, standard error; VF, vertebral fractures*.

* *P < 0.05*.

*Bold values are indicated for P < 0.05 (statistically significant). Italic values are indicated for all P-values*.

## Discussion

According to our findings, the overall VF incidence after childhood ALL treatment was 18.7%, which was a substantial proportion in this particular population. Out of various medical conditions causing secondary osteoporosis, our findings have underscored when VF have occurred in children undergoing ALL treatment, children with VF could compromised height, as well as greater height decline, than those without VF at the end of therapy. Older age of ALL diagnosis, greater dosage of GC, and lower height status contributed to the presence of VF. VF incidence was lower, while VF triggering age was older in our study compared to a former Canadian study (8). Although our study was conducted in similar age group, factors including ethnicity, discrepancy in treatment and follow up scheme could have affected the outcome. Compared to younger children with smaller skeletal dimension, more rapid and severe osteotoxic effect of using higher GC dosage might have contributed to higher VF incidence. Although both physical and mental recovery after TC in childhood ALL survivors remain as much a challenge as relapse-free survival, skeletal recovery is also implicated, as indicated by enhanced BMD accrual or healed fractures. Because the risk factors associated with VF incidence in the present study mostly cannot be manipulated, VF detection by routine surveillance throughout childhood ALL treatment, along with early intervention is recommended to prevent compromised growth.

Non-traumatic VF is a common skeletal manifestation in children with ALL that can occur at any time during the course of the disease, from the point of diagnosis to long periods following treatment (16). The rate of VF occurrence in children with ALL is increased more than 2000 times that in the general population (17). Therefore, routine vertebral assessment is critical, since moderate or even severe VF might develop asymptomatically. Cytokines released by leukemic cells during the leukemic process cause osteoclast-mediated bone resorption, and GC (prednisolone and dexamethasone), the mainstay drug for ALL treatment, exerts a strong altering effect on bone remodeling by the loss of bone volume during ALL treatment (16, 18). Our results also indicate that VF incidence is associated with the treatment duration and accumulated GC dose. Since these treatment-associated factors are standard, physicians should focus more on routine VF surveillance, followed by provision of appropriate nutritional support and physical exercise throughout the course of ALL treatment to minimize the risk of VF incidence.

Reduction in linear growth is a common complication of ALL treatment. Patients enrolled in the Canadian STeroid-Associated Osteoporosis in the Pediatric Population study showed 0.50 ± 0.40 and 0.40 ± 0.40 reductions in baseline height SDS in the first 6 and 12 months for boys and girls, respectively, followed by subsequent recovery. The accumulated daily GC dose played a major role in the observed height decline (11). Consistent with previous results, children with VF in our study demonstrated greater height decline during the treatment period and the presence of VF was a triggering factor of height decline. Hence, the presence of VF may be a major indicator of height loss. Because of the continuous bone growth in children, reduced height growth induced by VF could impair the elongation of growth and puberty and the attainment of peak bone mass (19). According to Suenobu et al., the critical point of reduced height velocity was during the interval between pretreatment and maintenance therapy, while height recovery was detected from the beginning of maintenance therapy until the end of treatment (20). As demonstrated in our study, further height decline was not observed after TC in both patient groups.

BMI was increased in children after ALL treatment and further increased in children with VF, while it decreased in those without VF at 12 months after TC. Increase in BMI was more prominent in children with VF than in those without VF during the 12-months period after TC. Given the lower degree of change in weight compared with height, BMI was relatively increased in children with VF during the treatment period. Factors such as nutritional deficiencies and immobility during the treatment period are considered possible causes of the weight and height reduction in children with VF. Weight gain is a worrisome late effect in childhood ALL survivors, which usually starts soon after the commencement of therapy, and is a common problem that is associated with the risks of cardiovascular and metabolic comorbidities (21). Based on the findings from this study, in children with VF, early prevention is recommended not to end up being overweight or obese, which would eventually reduce the risk of developing obesity-related metabolic conditions.

The clinical definition of osteoporosis in children includes under-mineralized bone, increased bone fragility with associated changes in bone microarchitecture, and increased risk of fracture (22). Recent guidelines proposed by the International Society for Clinical Densitometry suggest that pediatric osteoporosis is defined as follows: (1) one or more VFs in the absence of local disease or high-energy trauma or (2) a clinically significant fracture history (two or more long bone fractures by age 10 years or three or more long bone fractures at any age up to 19 years) and BMD (spine or total body less head preferred) SDS ≤ −2.0 (23). DXA values are preferred, but they do not provide a definite indication because the diagnosis of pediatric osteoporosis does not solely depend on densitometric criteria. Moreover, DXA scans appear to have limitations such as lack of robust reference databases for younger children, lack of clinical outcome related to densitometric measurements, and inaccuracies in measurement due to changes in body size and composition associated with growth (24). Since measuring BMD in younger children is challenging by using DXA, detection of VF should prioritize bone mass measurement, and D-L spine radiograph appears as an easier option for VF assessment. None of the children with VF complained of any signs of bone pain. Children with VF might be overlooked from among those with normal lumbar BMD values; therefore, spine radiographs and DXA scans should be performed concurrently to compensate each other for a reasonable interpretation of pediatric osteoporosis. Considering the VF incidence, skeletal assessment by D-L spine radiographs and DXA scans along with growth assessment are needed in children at pre- and post-treatment in order to determine the bone health status and investigate any treatment induced skeletal defect, and the surveillance might be necessary in the middle of the treatment, especially for those who have shown height decline. Early detection of VF could lead to early intervention of calcium and vitamin D as well as early administration of bisphosphonate which would possibly reduce further risk of fracture. The VF occurrence could also influence uncritical modification or even significant reduction of glucocorticoid dosage.

Although this study investigated the auxological profiles of children with ALL from the time of ALL diagnosis up to 4 years after treatment initiation, our findings should be interpreted with caution. First, D-L spine radiographs were not performed in children at LD. The radiographs could have enabled the exclusion of patients with pre-existing VF or other primary skeletal diseases. Second, the present data are observational, and the cohort size is small, which made comparisons in patient characteristics statistically infeasible. Note that the significant associations and suggested risk factors are not representative of a larger childhood ALL population. Also, the study did not include patients who relapsed or died prior to treatment completion. Patients with such early events most likely had higher risk ALL, and the exclusion of these patients may have had an impact on the overall incidence of VF. Third, although Genant semiquantitative assessment is an uncomplicated diagnostic tool for VF, visual disturbance or radiographic artifacts caused by lung parenchymal disease or patient movement during image acquisition may have been present, leading to over- or underdiagnosis of VF. Lastly, BMD measurements were performed in a limited number of children; thus, we were incapable of performing statistical analyses since the age- and sex-specific BMD normative data were obtained from a population of Korean children aged >10 years.

In conclusion, a substantial number of children experience asymptomatic VF after the completion of ALL treatment. Older age and lower height status at LD are potentially associated with VF incidence. Moreover, VF might adversely affect auxological status in childhood. Routine VF surveillance at pre- and post-treatment, is critical for the prevention of future VF incidence. Further long-term studies involving a large-scale cohort are needed to clarify the effect of VF on growth.

## Data Availability Statement

The raw data supporting the conclusions of this article will be made available by the authors, without undue reservation.

## Ethics Statement

The studies involving human participants were reviewed and approved by Institutional Review Board of the Catholic University of Korea, Seoul St. Mary's hospital. Written informed consent from the participants' legal guardian/next of kin was not required to participate in this study in accordance with the national legislation and the institutional requirements.

## Author Contributions

MA: study conception and sign. MA and SK: acquisition of data. MA, WC, JL, and BS: analysis and interpretation of data. NC, BC, MJ, and BS: drafting and revising the manuscript. All authors contributed to the article and approved the submitted version.

## Conflict of Interest

The authors declare that the research was conducted in the absence of any commercial or financial relationships that could be construed as a potential conflict of interest.

## Abbreviations

ALL: acute lymphoblastic leukemia
VF: vertebral fractures
LD: leukemia diagnosis
TC: treatment completion
BMD: bone mineral density
BMI: body mass index
SDS: standard deviation scores
LBM: Low bone mass
GC: glucocorticoids.

## References

[1] Malard F Mohty M. Acute lymphoblastic leukaemia. Lancet. (2020) 395:1146–62. 10.1016/S0140-6736(19)33018-1 32247396

[2] Howard SC Pui CH. Endocrine complications in pediatric patients with acute lymphoblastic leukemia. Blood Rev. (2002) 16:225–43. 10.1016/s0268-960x(02)00042-5 12350366

[3] Mostoufi-Moab S Ward LM. Skeletal morbidity in children and adolescents during and following cancer therapy. Horm Res Paediatr. (2019) 91:137–51. 10.1159/000494809 30481777 PMC6536370

[4] Ward LM. Osteoporosis due to glucocorticoid use in children with chronic illness. Horm Res. (2005) 64:209–21. 10.1159/000088976 16227699

[5] Jin HY Lee JA. Low bone mineral density in children and adolescents with cancer. Ann Pediatr Endocrinol Metab. (2020) 25:137–44. 10.6065/apem.2040060.030 33017885 PMC7538298

[6] Yang HR. Updates on bone health in children with gastrointestinal diseases. Ann Pediatr Endocrinol Metab. (2020) 25:10–4. 10.6065/apem.2020.25.1.10 32252211 PMC7136502

[7] Ko A Kong J Samadov F Mukhamedov A Kim YM Lee YJ. Bone health in pediatric patients with neurological disorders. Ann Pediatr Endocrinol Metab. (2020) 25:15–23. 10.6065/apem.2020.25.1.15 32252212 PMC7136510

[8] Cummings EA Ma J Fernandez CV Halton J Alos N Miettunen PM. Incident vertebral fractures in children with leukemia during the four years following diagnosis. J Clin Endocrinol Metab. (2015) 100:3408–17. 10.1210/JC.2015-2176 26171800 PMC4909472

[9] Saraff V Hogler W. ENDOCRINOLOGY aND aDOLESCENCE: osteoporosis in children: diagnosis and management. Eur J Endocrinol. (2015) 173:R185–97. 10.1530/EJE-14-0865 26041077

[10] Michigami T. Skeletal mineralization: mechanisms and diseases. Ann Pediatr Endocrinol Metab. (2019) 24:213–9. 10.6065/apem.2019.24.4.213 31905439 PMC6944863

[11] Ma J Siminoski K Alos N Halton J Ho J Cummings EA. Impact of vertebral fractures and glucocorticoid exposure on height deficits in children during treatment of leukemia. J Clin Endocrinol Metab. (2019) 104:213–22. 10.1210/jc.2018-01083 30247635 PMC6291659

[12] Lee JW Kim SK Jang PS Jeong DC Chung NG Cho B. Treatment of children with acute lymphoblastic leukemia with risk group based intensification and omission of cranial irradiation: a Korean study of 295 patients. Pediatr Blood Cancer. (2016) 63:1966–73. 10.1002/pbc.26136 27463364

[13] Kim JH Yun S Hwang SS Shim JO Chae HW Lee YJ. The 2017 korean national growth charts for children and adolescents: development, improvement, and prospects. Korean J Pediatr. (2018) 61:135–49. 10.3345/kjp.2018.61.5.135 29853938 PMC5976563

[14] Genant HK Wu CY van Kuijk C Nevitt MC. Vertebral fracture assessment using a semiquantitative technique. J Bone Miner Res. (1993) 8:1137–48. 10.1002/jbmr.5650080915 8237484

[15] Yi KH Hwang JS Kim EY Lee JA Kim DH Lim JS. Reference values for bone mineral density according to age with body size adjustment in korean children and adolescents. J Bone Miner Metab. (2014) 32:281–9. 10.1007/s00774-013-0488-z 23832576

[16] Ahn MB Suh BK. Bone morbidity in pediatric acute lymphoblastic leukemia. Ann Pediatr Endocrinol Metab. (2020) 25:1–9. 10.6065/apem.2020.25.1.1 32252210 PMC7136509

[17] Ward LM Ma J Lang B Ho J Alos N Matzinger MA. Bone morbidity and recovery in children with acute lymphoblastic leukemia: results of a six-year prospective cohort study. J Bone Miner Res. (2018) 33:1435–43. 10.1002/jbmr.3447 29786884

[18] Pufall MA. Glucocorticoids and cancer. Adv Exp Med Biol. (2015) 872:315–33. 10.1007/978-1-4939-2895-8_14 26216001 PMC5546099

[19] Gayretli Aydin ZG Bideci A Emeksiz HC Celik N Doger E Bukan N. Assessment of bone turnover markers and bone mineral density in normal short boys. J Pediatr Endocrinol Metab. (2015) 28:1321–6. 10.1515/jpem-2014-0099 26197459

[20] Suenobu S Goto H Hirano N Sonoda T Izumi T Ihara K. Early recovery of height velocity in prepubertal children with acute lymphoblastic leukemia treated by a short intensive phase without cranial radiation therapy. J Pediatr Hematol Oncol. (2020) 42:271–4. 10.1097/MPH.0000000000001695 31842181

[21] Bruzzi P Predieri B Corrias A Marsciani A Street ME Rossidivita A. Final height and body mass index in adult survivors of childhood acute lymphoblastic leukemia treated without cranial radiotherapy: a retrospective longitudinal multicenter italian study. BMC Pediatr. (2014) 14:236. 10.1186/1471-2431-14-236 25245636 PMC4194356

[22] Boyce AM Gafni RI. Approach to the child with fractures. J Clin Endocrinol Metab. (2011) 96:1943–52. 10.1210/jc.2010-2546 21734001 PMC3135196

[23] Shuhart CR Yeap SS Anderson PA Jankowski LG Lewiecki EM Morse LR. Executive summary of the 2019 iSCD position development conference on monitoring treatment, dXA cross-calibration and least significant change, spinal cord injury, peri-prosthetic and orthopedic bone health, transgender medicine, and pediatrics. J Clin Densitom. (2019) 22:453–71. 10.1016/j.jocd.2019.07.001 31400968

[24] Pezzuti IL Kakehasi AM Filgueiras MT de Guimaraes JA de Lacerda IAC Silva IN. Imaging methods for bone mass evaluation during childhood and adolescence: an update. J Pediatr Endocrinol Metab. (2017) 30:485–97. 10.1515/jpem-2016-0252 28328530

